# From specificity to sensitivity: affective states modulate visual working memory for emotional expressive faces

**DOI:** 10.3389/fpsyg.2015.01297

**Published:** 2015-08-27

**Authors:** Thomas Maran, Pierre Sachse, Marco Furtner

**Affiliations:** Department of Psychology, University of InnsbruckInnsbruck, Austria

**Keywords:** visual working memory, emotion, face processing, stress, mood, context

## Abstract

Previous findings suggest that visual working memory (VWM) preferentially remembers angry looking faces. However, the meaning of facial actions is construed in relation to context. To date, there are no studies investigating the role of perceiver-based context when processing emotional cues in VWM. To explore the influence of affective context on VWM for faces, we conducted two experiments using both a VWM task for emotionally expressive faces and a mood induction procedure. Affective context was manipulated by unpleasant (Experiment 1) and pleasant (Experiment 2) IAPS pictures in order to induce an affect high in motivational intensity (defensive or appetitive, respectively) compared to a low arousal control condition. Results indicated specifically increased sensitivity of VWM for angry looking faces in the neutral condition. Enhanced VWM for angry faces was prevented by inducing affects of high motivational intensity. In both experiments, affective states led to a switch from specific enhancement of angry expressions in VWM to an equally sensitive representation of all emotional expressions. Our findings demonstrate that emotional expressions are of different behavioral relevance for the receiver depending on the affective context, supporting a functional organization of VWM along with flexible resource allocation. In VWM, stimulus processing adjusts to situational requirements and transitions from a specifically prioritizing default mode in predictable environments to a sensitive, hypervigilant mode in exposure to emotional events.

## Introduction

Day-to-day life social interactions are of dynamic nature and require a series of cognitive efforts in order to communicate successfully. To be able to follow the dynamic requirements of an interaction among conspecifics, it is crucial to memorize communicated content temporarily for a few seconds, even though attention is already focused on new content. In short, it is critical to preserve a representation of previous communication without maintaining current sensory stimulus presence. Efforts of visual working memory (VWM) are of particular importance, since we need to be aware of the affective expressions of our counterparts for the duration of an interaction in order to act and react adaptively (Frith, 2009). In fact, findings in developmental psychology provide proof of domain-specific development of face memory, but not face perception (Weigelt et al., 2014). Facial expressions of anger constitute a striking social signal and a cue pointing toward interpersonal danger. This expression conveys aggressive intentions, dominance, and an intention to approach, making the sender appear more mature (Marsh et al., 2005). There is ample evidence for an anger superiority effect in different cognitive domains. This effect describes the preference for attention-related processing of angry expressions or its typical mimic attributes and has consistently been proven for the general population as well as clinical populations (Hansen and Hansen, 1988; Öhman et al., 2001; Frischen et al., 2008; Armstrong and Olatunji, 2012; Shasteen et al., 2014). Recently, Lundqvist et al. (2014) postulated an arousal hypothesis of the anger superiority effect and were able to show impressively that both within-item and between-expression differences in emotional arousal determine detection efficiency during visual search. Apart from the mentioned prioritizing of anger expressions with regard to attentional processing, there is evidence showing higher sensitivity for anger expressions in VWM in comparison to neutral or happy facial expressions (Jackson et al., 2009). Due to the fact that emotional facial expressions change rapidly, representations of emotional signals of the social counterpart are essential in order to control interactions. Particularly when the sender communicates potentially aggressive intentions by expressing anger, identities tagged with threat mobilize more resources and are thus more easily remembered than other emotional facial expressions (Sessa et al., 2011; Jackson et al., 2014). From a functionalist perspective, natural selection is believed to have favored perceptual bias toward environmental cues associated with danger as defense against threat of conspecifics (Haselton and Nettle, 2006). The ascription of aggressive intentions should thereby increase with growing uncertainty about the social counterpart and the context (Duntley, 2005).

Unlike investigating the processing of emotional expressions in an isolated and de-contextualized manner, in everyday life we interact with faces on bodies in specific natural and social environments (Barrett et al., 2011; Wieser and Brosch, 2012). Specific contractions of facial muscles communicate basic behavioral tendencies (Schyns et al., 2007). Drawing conclusions to more specific emotional states on the other hand is only possible in consideration of the context (Barrett, 2006). There is broad empirical evidence concerning the influence of stimulus-based context on perception of emotions (Barrett et al., 2011). It has been demonstrated that bodies (Aviezer et al., 2012; Kret et al., 2013; Van den Stock and de Gelder, 2014), pictures (Righart and de Gelder, 2008; Van den Stock et al., 2014), or voices (Van den Stock et al., 2007) presented parallel to faces influence face processing, as well as activation of implicit stereotypes or explicit information about the person shown (Carroll and Russell, 1996; Lucas et al., 2011; Neta et al., 2011; Schwarz et al., 2012) and cultural context (Elfenbein et al., 2007; Masuda et al., 2008). Additionally, stable traits and temporary states of the observer as perceiver-based context exert influence over the perception of emotions (Fox and Zougkou, 2011). Moreover, not only face perception, but also face recognition is modulated by affective context. Recent findings suggest that emotional scenes as immediate physical context of faces lead to less elaborate processing of a face, resulting in decreased facial recognition memory (Van den Stock and de Gelder, 2012). Despite clear evidence supporting the influence of a series of context variables on face processing, so far there are no studies investigating the impact of affective states on processing of socio-emotional signals, particularly the anger superiority effect.

Activation of fundamental motives by exposure to environmental cues associated with self-protection, mating or immediate physiological needs cause affective states high in motivational intensity (Adolphs, 2010; Lang, 2010; Lang and Bradley, 2010, 2013; LeDoux, 2012). Such a motivational state results in an indiscriminate hypervigilance along with enhanced sympathetic arousal (Berridge and Waterhouse, 2003; Löw et al., 2008; Lang and Bradley, 2010), supporting appetitive or defensive reactions to current situational challenges (Bradley et al., 2012; Pichon et al., 2012; Miskovic and Keil, 2014). This adaptive change in cognitive processing mode featuring unspecific, increased attention and sensory processing (Berridge and Waterhouse, 2003; Phelps et al., 2006) enables the organism to generate a prompt representation of the environment at the expense of an increasing probability of false alarms (Payne et al., 2002; Duncko et al., 2009). Thereby, affective states act as superordinate mechanisms that orchestrate cognition and behavior in a functional, specific mode (Al-Shawaf et al., 2015; Lench et al., 2015) and constitute an influential perceiver-based context for cognitive processing of environmental cues (Mather and Sutherland, 2011; Dolcos et al., 2014). In fact, experimentally induced, highly arousing affective states alter working memory performance (Shackman et al., 2006; Luethi et al., 2008; Schoofs et al., 2008, 2009; Duncko et al., 2009) and lead to a rapid extraction and subsequent remembering of the gist of an event (Kensinger et al., 2007; Qin et al., 2012). Recent evidence suggests that the motivational intensity (Larson and Steuer, 2009; Gable and Harmon-Jones, 2010a) of an affective state represents a crucial dimension, which accounts for the modulation of attentional and mnemonic processing by broadening or narrowing of the cognitive scope (Harmon-Jones and Gable, 2009; Gable and Harmon-Jones, 2010b,c; Harmon-Jones et al., 2011, 2012).

Jackson et al. (2009) suggest that the anger superiority effect in VWM might arise from a tuned integration of the visual information of an angry looking face, resulting in the storage of a greater quantity of information. This enhanced representation of angry faces is stimulus-driven, arising from the immanent emotional content conveyed by the angry looking face. Furthermore, the authors were able to show that the enhanced storage of threat-related faces in VWM is abolished when faces are inverted, a manipulation that is meant to disrupt holistic face processing (Van Belle et al., 2010). In fact, faces (Richler and Gauthier, 2014) and most likely also emotional facial expressions (Tanaka et al., 2012) are being processed in a holistic way. In addition to face inversion, recent findings suggest that negative affective states abolish holistic face perception by narrowing cognitive scope and thereby promoting a more feature-based processing of faces (Curby et al., 2012). Focusing on details or features rather than the holistic percept of an affective salient cue is supposed to be a form of emotion regulation (Todd et al., 2012a) by reducing the extraction of the immanent emotional content of a cue (Everaert et al., 2014).

Taken together, evidence supports the notion of a strong influence of affective states on working memory and face processing. Affective states of high motivational intensity lead to a switch from a default mode to a hypervigilant mode of cognitive processing (Berridge and Waterhouse, 2003) and thereby to a narrowed cognitive scope in attention and memory (Gable and Harmon-Jones, 2010a). Holistic perception of emotionally expressive faces and thus the extraction of the immanent emotional content of an expression is abolished by a state-induced narrowed scope and thereby feature-based processing (Curby et al., 2012). Therefore, we predict a modulation of VWM for emotional expressive faces by affective states of high motivational intensity. In a low arousal affective state, default mode processing promotes increased specificity for affective salient cues, resulting in enhanced storage of emotion-laden stimuli, such as angry looking faces (Jackson et al., 2009). In an affective state of high motivational intensity, hypervigilant mode processing promotes an unspecified sensitivity for all stimuli by less holistic encoding of emotional salient cues and thereby reduced extraction of their emotional content (Everaert et al., 2014). Thus, we expect emotionally expressive faces to be equally represented in VWM without any specific prioritization, resulting in an affect-induced alignment for all encoded stimuli. In two experiments, the present study aims to empirically test these predictions. In Experiment 1, participants performed a VWM task featuring emotionally expressive faces in two blocks using a counterbalanced within-subject design. In one block, they were confronted with highly arousing, negatively valenced scenes in order to induce an affective state high in motivational intensity, whereas in the other block, participants were exposed to neutral pictures. In Experiment 2, in the same within-subject design, we used highly arousing, positively valenced scenes instead of aversive scenes. We predict an affect-induced alignment in VWM for all emotionally expressive faces, whereas in the neutral condition, a specifically enhanced storage of angry looking faces should occur. The same performance patterns are expected for both experiments, whereby motivational intensity instead of valence would be confirmed as the crucial dimension in modulating VWM processing mode.

## General Method

### Face Stimuli

The experimental task to measure VWM performance was comprised of colored pictures of eight Caucasian, male, adult individuals of the NimStim set of facial expressions (Tottenham et al., 2009). Each of those individuals displayed an angry, anxious, happy, and neutral facial expression. The availability of the four emotional facial expressions with closed mouths was an essential criterion when selecting the identities. Higher salience due to increased local contrast might distort recognition of the images when showing open mouths and thus is considered a confounding factor. For all pictures, any parts not belonging to the face were removed to ensure the stimuli depicted the face alone in a nearly oval shape. The shape was identical for all eight faces and all four emotional expressions, including all relevant traits of a face. Luminance and color were adjusted. The stimuli were edited using Adobe Photoshop CS6. A face stimulus subtended 2,20°×2,96° of visual angle, corresponding to a size of 102 × 137 pixels in this setting. All faces were male, since male angry faces are perceived as more threatening and dominant than female angry expressions (Öhman et al., 1985; Daly and Wilson, 1988; Becker et al., 2007).

Arousal caused by face stimuli plays a key role in terms of attentional bias toward threatening faces in visual search protocols (Lundqvist et al., 2014). Considering this finding, participants rated the utilized faces of the NimStim set after Experiment 1 (*n* = 24, 12 female, 12 male). Using E-Prime software (Version 2.0; Psychology Software Tools, Pittsburgh, PA, USA; Schneider et al., 2012), thirty-two faces were presented in the center of the screen in random order. At first, participants matched faces with presented emotional expressions (angry, fearful, happy, neutral) in a forced-choice response format and subsequently rated the emotionally expressive faces on a five-point scale according to their intensity (1 for weak, 5 for strong). For emotion recognition, a repeated-measures analysis of variance (ANOVA) showed a significant difference between the emotional expressions, *F*(3,69) = 5.668, *p* = 0.002, η^2^ = 0.198 (hit rates: angry 94%, fearful 86%, happy 96%, neutral 85%). Bonferroni corrections applied to *post hoc* comparisons revealed that happy expressions were more easily identified than anxious (*p* = 0.009) and neutral faces (*p* = 0.027). However, data analysis indicated no significant differences in perceived intensity of emotional expressions *F*(3,69) = 0.136, *p* = 0.938.

### Scenes

**FIGURE 1:**
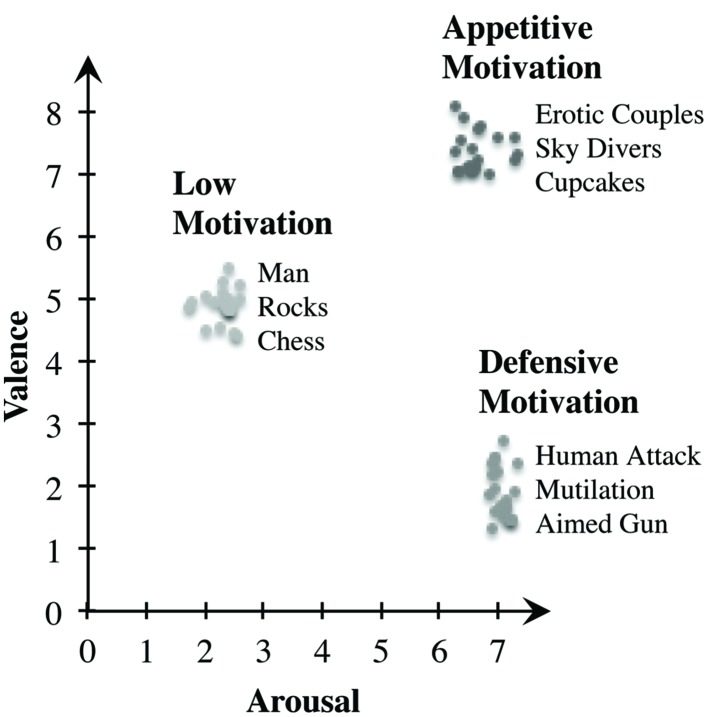
**Ratings of the scenes from the International Affective Picture System (IAPS).** The *y*-axis displays mean pleasure, the *x*-axis displays mean arousal. Ratings of the 20 images per condition are noted, as well as three examples of each presented category. Along the trajectories of appetitive and defensive motivation, the selected pictures are positioned at the end of the respective line (Lang et al., 2005).

In the study at hand, two experiments should induce either a state of unpleasant affect (Experiment 1), or a state of pleasant affect (Experiment 2), both high in motivational intensity. For this purpose, we selected a total of 60 photographs of the International Affective Picture System (IAPS; Lang et al., 2005). IAPS contains extensive ratings along the dimensions of valence and arousal. Both dimensions provide a two-dimensional affective space with two clear trajectories: Activation of an appetitive motivation system with increasing valence (pleasant affect) and increasing arousal as well as activation of a defensive motivation system with decreasing hedonic valence (unpleasant affect) and increasing arousal (Bradley et al., 2001). Photographs of erotic scenes or exciting sports stimulate pleasant affect and appetitive motivation, whereas mutilations, weapons, or violent scenarios cause unpleasant affect and defensive motivation (Bradley and Lang, 2007; Lang and Bradley, 2010). In Experiment 1, we selected twenty scenes of negative valence (unpleasant) and high arousal (e.g., mutilations, human attack), as well as twenty low arousing pictures of medium, therefore neutral valence (e.g., day-to-day situations, chess) as a control condition. In contrast, Experiment 2 features another 20 scenes with positive valence (pleasant) and high arousal (e.g., erotic couples, skydivers), as well as the same twenty control pictures as in Experiment 1. Along the dimensions of appetitive and defensive motivation (Bradley et al., 2001), the selected images portray the extreme ends of the two trajectories (see **Figure 1**). An ANOVA revealed significant differences in perceived valence between the three groups, *F*(2,57) = 1279.682, *p* < 0.001, multiple comparisons indicating that the group of appetitively arousing photographs was perceived as being more pleasant than the defensively arousing photographs (*p* < 0.001) and the control scenes (*p* < 0.001). Control scenes were also rated as being more pleasant than the group of negatively arousing pictures (*p* < 0.001). Further data analysis showed significant differences in perceived arousal when looking at the groups of pictures *F*(2,57) = 2125.581, *p* < 0.001. The group of unpleasant pictures showed higher arousal ratings than pleasant (*p* < 0.001) and neutral images (*p* < 0.001). Likewise, pleasant photographs led to higher arousal than neutral control pictures (*p* < 0.001). Altogether, results gained by analyzing the IAPS ratings confirm the suitability of the chosen IAPS scenes for inducing affective states with the intended high intensity and motivational direction.

### VWM Task for Emotional Faces

**FIGURE 2:**
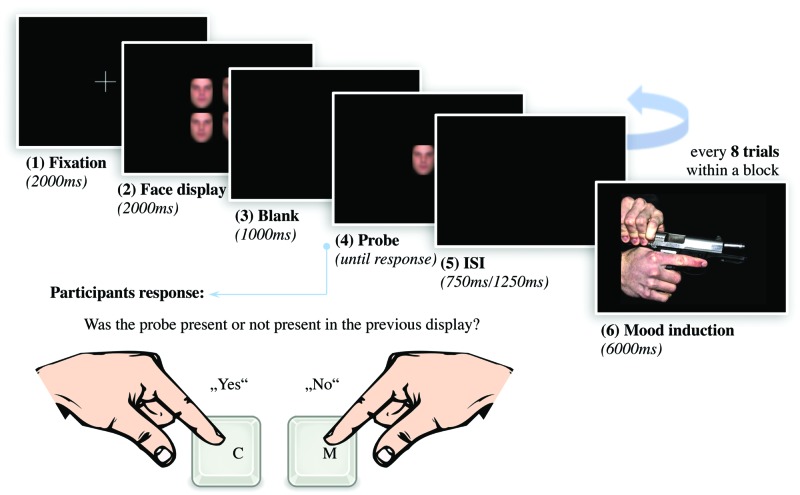
**Structure of the experimental task to register VWM for emotionally expressive faces with experimental mood induction.** After presenting a fixation cross for 2000 ms, we showed a 2 × 2 array of four faces displaying the same emotional expression for 2000 ms. After a retention interval of 1000 ms, a single test face was presented. Participants assessed whether the test face was part of the previous array or not by pressing two buttons (C = yes, M = no). To manipulate mood as perceiver-based context, we presented an IAPS scene for 6000 ms after eight trials each (The picture in the figure originate from internet databases of unrestricted use).

In this experimental task (see **Figure 2**), a blank screen interval of 2000ms followed a 2 × 2 array of four faces to be remembered, also for 2000 ms. The array of the faces subtended a visual angle of approximately 4,63° × 6,14°. To eliminate confounding effects by selective attention, all four faces displayed the same expression; either angry, anxious, happy, or neutral. Following the four-face display, a blank screen of 1000 ms was presented as retaining interval. Afterward, a single test face was presented and the participants were instructed to decide whether the said test face has been part of the previous face array or not. The target stimulus showed the same emotional expression as the stimuli during the encoding phase. The face remained on the screen until participants responded. Since the identity of the face was asked to be remembered, the emotional expression was irrelevant to complete the task. Every face was presented only once in a four-face array and both the combination of the respective faces and the order of the arrays were randomized. Also, the emotional expressions displayed by all four faces were randomized across every block. For each emotional expression within an experimental block, 40 trials were presented. In one half of the trials, the test face was part of the preceding array, whereas in the other half it was not. Including four emotional expressions of 20 “yes-trials” and 20 “no-trials” each, an experimental block consisted of a total of 160 trials. Each face identity was presented with equal frequency in each block. Participants completed one block with neutral images and one with arousing images. Both blocks contained the same four-face displays, again in randomized order within each block. In order to prevent the application of verbal memory strategies, the phonological loop in working memory was occupied by repeated whispering of alternating letter pairs (Curby and Gauthier, 2007). In Experiment 1, the hit rate for letters was at 99%, in Experiment 2, 98% of the queries were answered correctly. In both experiments, the hit rate of the participants was at least 80%.

### Experimental Procedure

A within-subject design was used in which participants completed two blocks of the VWM task for emotionally expressive faces. To manipulate affective states experimentally, within each block, we presented twenty IAPS images for 6000 ms, following every eight trials of the VWM task (see **Figure 2**). Before and after the IAPS image, a blank screen was displayed for 2000 ms. Using a counterbalanced within-subject design, one half of participants faced highly arousing images followed by neutral control scenes, whereas the other half saw neutral images first, followed by arousing ones. Images of mutilations or violence (Experiment 1) induce an acute affective state with defensive behavioral preparedness, whereas scenes of erotica or adventure (Experiment 2) lead to an affective state high in appetitive motivational intensity (Bradley et al., 2001). Presenting such affect-inducing scenes prompts a perceiver-based context for the subsequent tasks. A change in performance between the affective and control condition is therefore traceable to the induction of defensive affect high in motivational intensity.

In order to induce affective states of high motivational intensity, a stimulus situation must be perceived as novel, unpredictable, and trigger a feeling of loss of control (Lupien et al., 2007). IAPS, as used in this design, is in accordance with these criteria. Previous studies relating to contextual influence of photographs on emotion processing presented the images as immediate, parallel context (de Gelder et al., 2006; Righart and de Gelder, 2006, 2008; Van den Stock and de Gelder, 2012; Van den Stock et al., 2014). In contrast to those findings, in our experiments we distributed IAPS images scattered throughout the duration of the experimental task before completion of a fixed number of trials. This should vary the affective state of the observer experimentally instead of varying the physical context of the faces. Since the effects of experimental mood induction decrease with increasing task duration (Schoofs et al., 2008), placing IAPS scenes within the blocks should ensure a continuous mood induction for the duration of the experimental task. The blocks were separated by a 5 min pause. Additionally, using this design to induce an affective perceiver-based context allows attributing effects on VWM performance patterns to the mood manipulation procedure, instead of emotion-induced, interfering orienting responses (Bradley et al., 2012) as triggered by scenes and bodies presented as immediate, parallel stimulus-based context (Van den Stock and de Gelder, 2012).

To check the effectiveness of the mood induction, we registered participants’ affect at the beginning, between blocks of the experimental task, and upon completion thereof by using the “Positive and Negative Affect Schedule” (PANAS; Watson and Clark, 1988; German translation by Krohne et al., 1996) as well as the State-Scale of the “State-Trait Anxiety Inventory” (STAI; Spielberger et al., 1970; German translation by Laux et al., 1981). Both scales allow capturing current mood by an evaluation of a series of words which describe various feelings.

We developed the experimental task using E-Prime software (Version 2.0; Psychology Software Tools, Pittsburgh, PA, USA; Schneider et al., 2012) and presented it on a Samsung 943BM monitor (32-bit true color; resolution 1280 × 1024 pixels, refresh rate = 60 Hz). Considering the varying responsiveness of an organism for psychological stressors over the course of the day (Kudielka et al., 2004), we conducted all experiments in the afternoon.

### Data Analysis

The key variable in both experiments is the performance of VWM for emotionally expressive faces. Change detection performance was quantified using d-prime (*d*′) as a measure of sensitivity according to signal detection theory (Macmillan and Creelman, 1991). We estimated *d*′ via average hit rate and false-alarm rate. For this purpose, we initially calculated the probability of correct match responses (hits) and incorrect match responses (false alarms) for each emotion and condition separately. The difference of the z-transformed hit rate and false-alarm rate results in sensitivity: *d*′ = z(*H*) – z(*F*). Corrections for extreme values in hit rates or false alarms were applied. Rates of 0 were replaced with 0.5/*n*, and rates of 1 were replaced with (*n* – 0.5)/*n, n* being the number of signal or noise trials (Stanislaw and Todorov, 1999). Sensitivity for recognizing the target stimulus was calculated separately for each participant concerning each emotional expression and each mood condition.

To examine the interaction between facial expression and mood, repeated-measures ANOVAs were applied to the rates of hits, rates of false alarms and *d*′ with emotional expression (angry, anxious, happy, neutral) and mood condition (neutral, arousing) as within-subject variables. Additional repeated-measures ANOVAs were conducted separately for each mood condition with facial expression as within-subject factor (angry, anxious, happy, neutral) in order to examine differences between emotional expressions. Degrees of freedom were corrected in case of deviance from sphericity (Greenhouse–Geisser). To locate differences between facial expressions, we applied Bonferroni corrected *post hoc* multiple comparisons. Comparisons between mood conditions (neutral, arousing) for each emotional expression separately were computed using paired-samples *t*-tests. Alpha levels were set at 0.05.

## Experiment 1

In Experiment 1, we examined the influence of highly defensive affect on sensitivity for emotionally expressive faces in VWM. To manipulate mood experimentally during the emotional block, affectively arousing, unpleasant images were displayed while participants completed the VWM task. The order of both blocks was counterbalanced across participants. For all 24 participants, we examined the self-reported effects of mood induction.

### Participants

All participants were undergraduate students and received research credits for participating in the experiments. All had normal or corrected-to-normal vision. None of the participants indicated suffering from or having first-degree relatives with diagnosed psychiatric conditions, being under the influence of psychoactive substances or psychopharmacological treatment, or having sustained a severe head injury in their lives (self-report). Three participants in Experiment 1 were excluded from the study because their performance was not better than chance. The results of 24 participants (12 females, 12 males; *M* = 22,54 years, SD = 3,18; the youngest participant being 19, the oldest 33 years old) were included in the data analysis. Three of the participants were left-handed. Ethics approval was received from the Ethics Committee of the University of Innsbruck and participants provided informed consent.

### Results

#### Mood Induction

**FIGURE 3:**
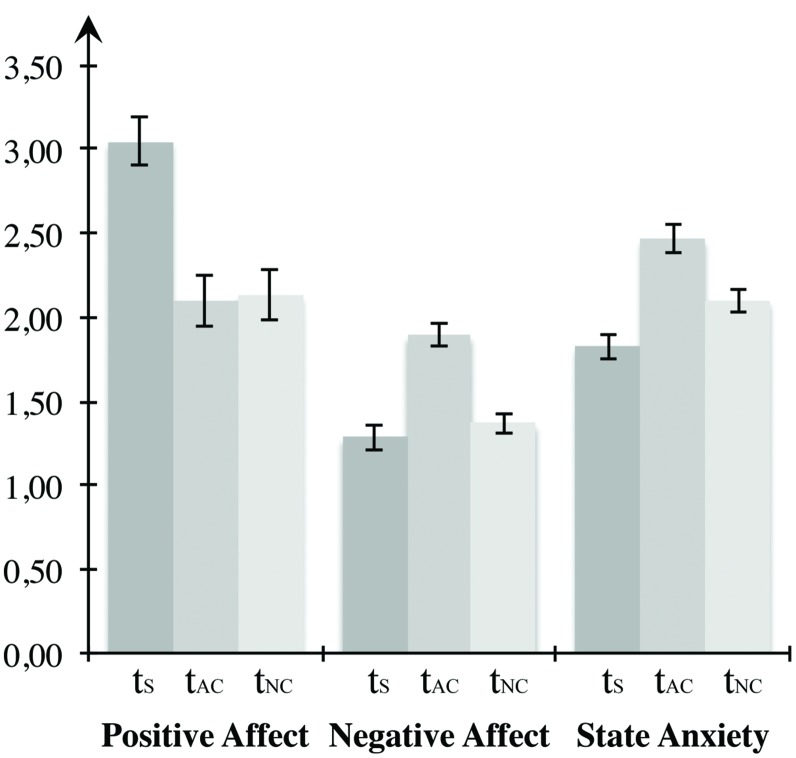
**Mood induction for Experiment 1.** The first two bar groups form the PANAS scores for positive and negative affect, the last bar group represents the STAI score for state of fear. All three states were measured before the first block, between the first and second block, as well as following the end of the last block. When conducting the experiment, presentation of the blocks was counterbalanced across participants, whereas here, *t*_S_ depicts the first measurement, *t*_AC_ the measurement after the affective block, and *t*_NC_ the measurement after the neutral block. Error bars depict ±1 SEM.

To analyze the self-report data on mood induction, we arranged the measurements in a uniform sequence according to the counterbalanced blocks (see **Figure 3**). Statistical manipulation checks confirmed that the mood induction procedure was effective. Repeated-measures ANOVAs indicated significant differences between the three times of measurement regarding both positive affect, *F*(2,46) = 39.333, *p* < 0.001, η^2^ = 0.631, as well as negative affect, *F*(2,46) = 13.868, *p* < 0.001, η^2^ = 0.376, and state anxiety, *F*(2,46) = 20.362, *p* < 0.000, η^2^ = 0.470. Multiple comparisons using Bonferroni corrections demonstrated that positive affect was most pronounced in the beginning, differing significantly from both the value after the negative arousal block (*p* < 0001) and after the neutral block (*p* < 0.001). However, no change was observed between measurements after the negative and the neutral block (*p* = 1.000). Negative affect at the time after the negative block differed significantly from the first time of measurement (*p* = 0.002) and the measurement after the low arousal block (*p* = 0.002), but there was no significant change between the initial measurement and the measurement after the neutral block (*p* = 0.921). Presenting highly arousing IAPS images led to a significant increase of state anxiety, compared both to the measurement before the task (*p* < 0.001) as well as the measurement after the low arousal block (*p* = 0.003). Furthermore, participants displayed significantly elevated anxiety in the measurement after the neutral condition compared to the first measurement (*p* = 0.002). Since participants performed at least one block of the VWM task with a duration of 21 min before the self-report assessment after the neutral block, the decrease of positive affect and the increase of state anxiety after the neutral block compared to the input measurement might be a consequence of exhaustion due to the challenging task demands. In total, the self-report data confirm the effectiveness of mood induction in the predicted direction. Both negative affect and anxiety increased compared to the control condition due to the high arousal IAPS images.

#### Negative Affective State and VWM for Emotional Faces

**FIGURE 4:**
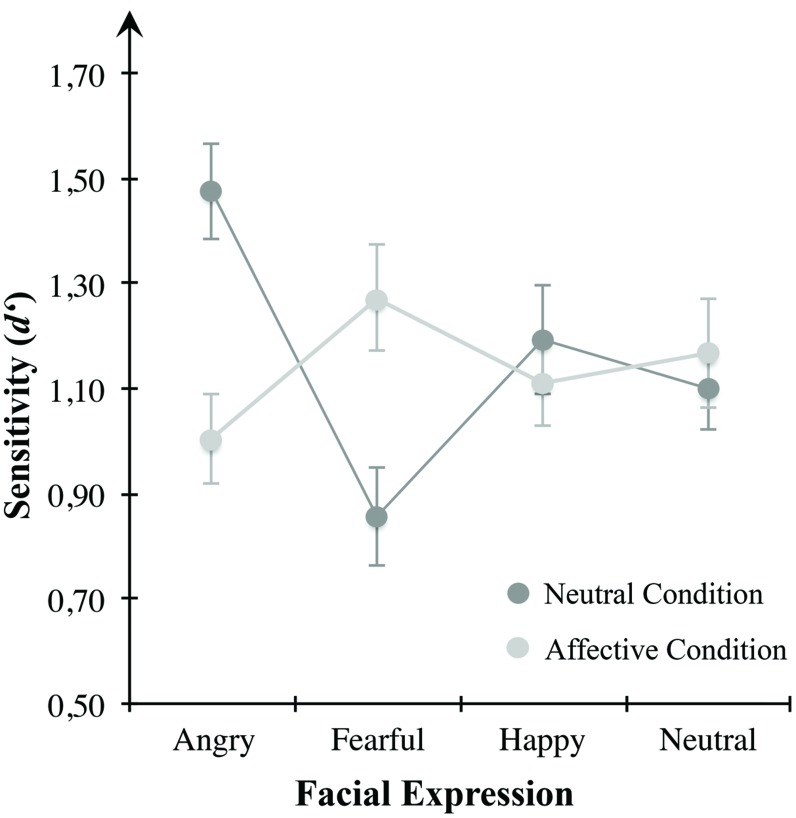
**Mean *d*′ scores for the mood conditions of Experiment 1.** Sensitivity in VWM for emotionally expressive faces is depicted for the affective condition high in defensive motivation (bright gray circles) and the neutral condition (dark gray circles). Error bars depict ±1 SEM.

To test affect-induced alterations in VWM for emotional expressive faces, we applied repeated-measures ANOVA for sensitivity *d*′ (see **Figure 4**) using emotional expression (angry, anxious, happy, neutral) and mood condition (neutral, negatively arousing) as within-subject variables. Results indicated a significant interaction effect between both within-subject factors *F*(3,69) = 10.364, *p* < 0.001, η^2^ = 0.311. However, there were no main effects of the within-subject variables of emotional expression across both mood conditions, *F*(3,69) = 1.928, *p* = 0.133, or mood condition across all presented emotional expressions, *F*(1,23) = 0.090, *p* = 0.767. These results indicate a change of sensitivity *d*′ for different emotional expressions depending on mood condition. **Table 1** describes group means for sensitivity, illustrated in **Figure 4**.

**Table 1:** Mean *d*′ scores for the mood conditions of Experiment 1.

	Neutral			Negative arousal		
Expression	Hit rates	False alarms	Sensitivity *d*′	Hit rates	False alarms	Sensitivity *d*′
Angry	0.73 (0.03)	0.24 (0.03)	1.48 (0.09)	0.67 (0.03)	0.32 (0.03)	1.00 (0.08)
Fearful	0.66 (0.03)	0.36 (0.04)	0.86 (0.09)	0.70 (0.03)	0.28 (0.03)	1.27 (0.10)
Happy	0.75 (0.03)	0.36 (0.04)	1.19 (0.10)	0.75 (0.02)	0.37 (0.04)	1.11 (0.08)
Neutral	0.67 (0.03)	0.29 (0.03)	1.10 (0.08)	0.66 (0.04)	0.28 (0.05)	1.17 (0.10)

*SE in parentheses.*

To examine VWM performance in the neutral condition compared to the negatively arousing one, we conducted separate repeated-measures ANOVAs and Bonferroni corrected *post hoc* multiple comparisons for each mood condition. In the neutral condition, the ANOVA revealed a significant main effect of the within-subject variable emotional expression, *F*(3,69) = 12.940, *p* < 0.001, η^2^ = 0.360. Multiple comparisons indicated higher sensitivity for angry faces in comparison to anxious (*p* < 0.001), happy (*p* = 0.030), or neutral expressions (*p* = 0.013). Likewise, there was a significant difference between happy and anxious expressions (*p* = 0.045). Other multiple comparisons were non-significant. However, consistent with the postulated assumptions, there was no main effect of the presented emotional expression in the negative arousal condition, *F*(3,69) = 1.837, *p* = 0.149.

To further clarify how VWM for emotional faces changed between both mood conditions we applied planned paired-samples *t*-tests for differences between the two conditions within the respective emotional expression. Results showed a significant reduction of sensitivity for angry looking faces from the neutral to the affective condition *t*(23) = –4.216, *p* < 0.001. Vice versa, *d*′ was higher for anxious faces in the affective condition compared to the neutral condition, *t*(23) = 4.140, *p* < 0.001. There were no significant differences between the two mood conditions with respect to happy, *t*(23) = –0.691, *p* = 0.496, and neutral looking faces, *t*(23) = 0.531, *p* = 0.601.

Next we specified whether the effect of affective state on sensitivity *d*′ in VWM is driven by alterations of hit rates or false alarms. For this purpose we applied the same statistical testing on hit rates and false alarms as used to analyze sensitivity *d*′. The repeated-measures ANOVA for hit rates using emotional expression and mood conditions as within-subject variables did not indicate a significant interaction, *F*(3,69) = 1.896, *p* = 0.138, and no significant main effect of the mood conditions, *F*(1,23) = 0.112, *p* = 0.741. However, there was a significant main effect of emotional expression, *F*(3,69) = 5.267, *p* = 0.002, η^2^ = 0.186. Multiple comparisons using Bonferroni corrections revealed a significantly higher hit rate for happy expressions compared to anxious (*p* = 0.009) and neutral (*p* = 0.005) expressions. Within each emotional category, paired-samples *t*-tests showed no significant differences between the neutral and the negative arousal condition. Considering the neutral condition separately, another significant main effect is revealed, *F*(3,69) = 5.738, *p* = 0.001, η^2^ = 0.200, and likewise, happy faces obtained a higher hit rate in comparison to anxious (*p* = 0.005) and neutral expressions (*p* = 0.035). Angry faces showed slightly higher significant hit rates than anxious faces (*p* = 0.093). Also, there was a slightly significant main effect in the negative arousal condition, *F*(3,69) = 2.641, *p* = 0.056, η^2^ = 0.103, again the hit rate for happy faces being higher than for neutral expressions (*p* = 0.038).

In contrast to hit rates, data analysis of false alarm rates indicated a significant interaction effect between the within-subject variables emotional expression and mood condition, *F*(3,66) = 5.975, *p* = 0.001, η^2^ = 0.214, as well as a main effect of the emotional category, *F*(3,66) = 4.709, *p* = 0.005, η^2^ = 0.176. In contrast, no significant main effect of mood conditions was observed, *F*(1,22) = 0.009, *p* = 0.927. Multiple comparisons indicated higher false alarm rates for happy faces compared to angry expressions (*p* = 0.010) and neutral faces (*p* = 0.034). Furthermore, there was a main effect of emotional expression in the neutral condition, *F*(3,66) = 9.476, *p* < 0.001, η^2^ = 0.301. Anxious faces showed significantly higher false alarm rates than angry ones (*p* < 0.001) and slightly significant higher false alarm rates than neutral expressions (*p* = 0.078). Moreover, happy faces showed a higher false alarm rate than angry expressions (*p* = 0.004). There was also a significant main effect in the negative arousal condition, *F*(3,69) = 2.733, *p* = 0.050, η^2^ = 0.106. As in the neutral condition, happy faces showed the highest false alarm rate, this time compared to anxious (*p* = 0.028) and neutral expressions (*p* = 0.064). When comparing the differences between the mood conditions within the emotional expression, paired-samples *t*-tests revealed a slightly significant increase of false alarms for angry faces in the negative arousal condition, *t*(23) = –1.899, *p* = 0.071, as well as a significant decrease of false alarms for anxious faces, *t*(23) = 2.190, *p* = 0.039. No changes were observed for happy, *t*(23) = –0.306, *p* = 0.762, or neutral expressions, *t*(23) = 0.317, *p* = 0.754.

### Discussion

Experiment 1 demonstrates the effects of affective states high in defensive motivation (e.g., anxiety) on VWM for emotionally expressive faces. These results indicate an enhanced storage of angry expressions in VWM during a low arousing affective state (Jackson et al., 2009). By contrast, exposure to negatively arousing scenes leads to an equalized VWM performance for all emotional expressions by inducing a state high in defensive motivational intensity. This switch from specificity to sensitivity is driven by a change of tendency for false alarms (Payne et al., 2002; Duncko et al., 2009). Exposure to negatively arousing events led to an increase of false alarms for angry looking faces compared to the neutral condition, whereas that for anxious expressions decreased. Altogether, this results in an alignment of sensitivity *d*′ for all emotional expressions in VWM. The performance patterns show a clear switch from a specifically prioritizing processing mode that accounts for the angry-face specificity in VWM to an undiscriminating, hypervigilant processing mode promoting an equalized sensitivity for various expressive faces in VWM.

## Experiment 2

Recent findings provide evidence that motivational intensity of an affect and strongly related arousal are essential dimensions when modulating cognitive processes, as opposed to valence (Gable and Harmon-Jones, 2010b,c; Harmon-Jones et al., 2011; Mather and Sutherland, 2011, 2012). Thus, events that induce defensive behavioral readiness as well as events that lead to a change in appetitive readiness alter cognitive processing. Accordingly, switching from modes for angry-face specificity to generalized sensitivity for various expressive faces in VWM is also expected to occur in affective states high in appetitive motivational intensity (e.g., desire).

In Experiment 2, we examined the influence of highly appetitive affect on sensitivity for emotionally expressive faces in VWM. In contrast to Experiment 1, we added highly arousing, pleasant images in order to induce an affective state high in motivational intensity. We implemented the experiment the same way as Experiment 1. For this repeated-measures design, we also counterbalanced the order of the arousal block and the control block across participants. For 24 of all 30 participants, we assessed self-reported effects of mood induction.

### Participants

We recruited the sample for Experiment 2 from the same population, using the same incentive as in Experiment 1. One participant did not fulfill the self-reported inclusion criteria and was excluded. Two other participants were not taken into account for the data analysis of Experiment 2. The results of thirty participants (20 females, 10 males; *M* = 22,60 years, SD = 2,14; the youngest participant being 19, the oldest 28 years old) were utilized. Two of the participants were left-handed. Ethics approval was received from the Ethics Committee of the University of Innsbruck and participants provided informed consent.

## Results

### Mood Induction

**FIGURE 5:**
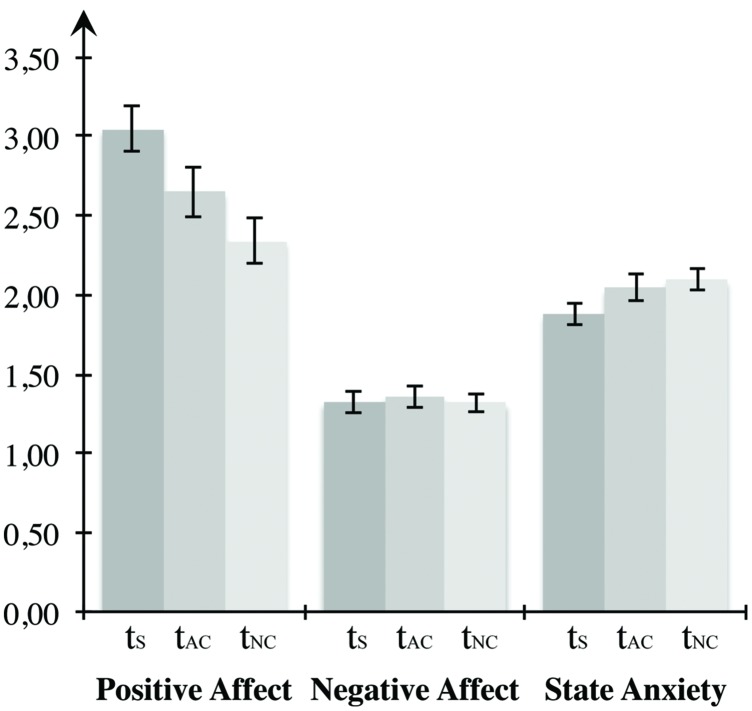
**Mood induction for Experiment 2.** The first two bar groups form the PANAS scores for positive and negative affect, the last bar group represents the STAI score for state anxiety, *t*_S_ depicts the first measurement, *t*_AC_ the measurement after the affective block, and *t*_NC_ the measurement after the neutral block. Error bars depict ±1 SEM.

Before testing the hypotheses for Experiment 2, we examined whether the mood induction procedure was effective by applying repeated-measures ANOVAs on self-report measures of the current affective state (see **Figure 5**). Statistical testing revealed significant changes with regard to positive affect, *F*(2,46) = 14.545, *p* < 0.001, η^2^ = 0.387. Results showed a decrease of positive affect between the first measurement and the measurement after the high arousal block (*p* = 0.039) and after the neutral condition (*p* < 0.001). Likewise, participants reported significantly higher positive affect after the positive arousal block compared to measurement after the control condition (*p* = 0.049). No differences were observed between time points with respect to negative affect, *F*(2,46) = 0.158, *p* = 0.855. Anxiety changed between measurements, *F*(2,46) = 4.948, *p* = 0.011, η^2^ = 0.177. Multiple comparisons revealed no difference between the measurement after the high arousal block and the initial measurement (*p* = 0.150) or the measurement after the neutral block (*p* = 1.000). However, the manifestation of state anxiety increased significantly between the first measurement and the measurement after the neutral condition (*p* = 0.019). Higher positive affect between the measurements after the emotional block in comparison to the neutral condition provides evidence for the effectiveness of the experimental mood induction. Both the significant decrease compared to the input measurement and the increase of anxiety between the input measurement and the measurement after the neutral block can be considered a consequence of the challenging face task processing.

### Positive Affective State and VWM for Emotional Faces

**FIGURE 6:**
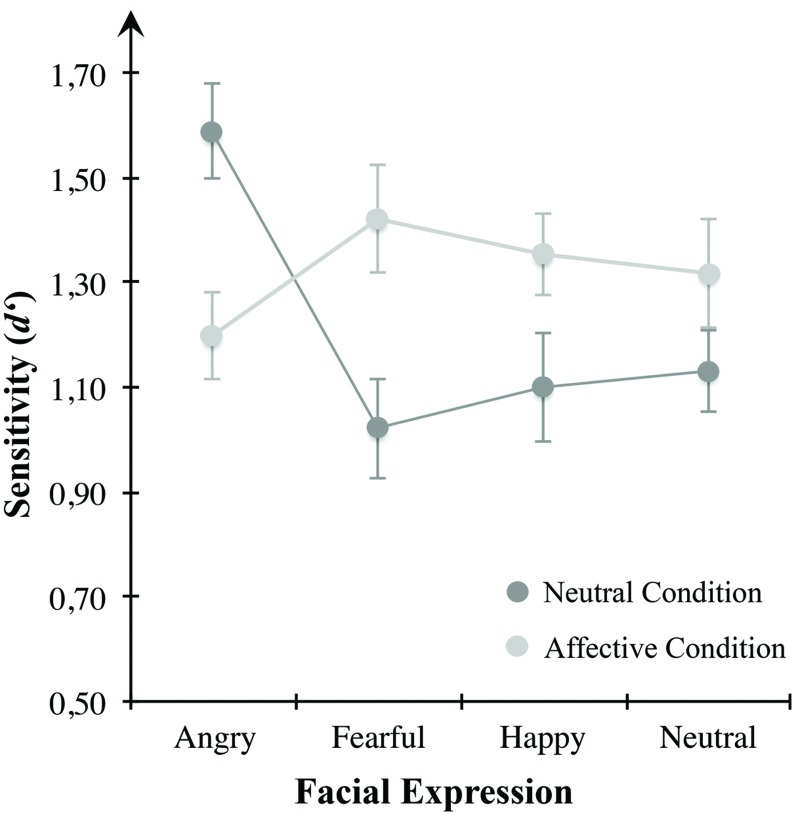
**Mean *d*′ scores for the mood conditions of Experiment 2.** Sensitivity in VWM for emotionally expressive faces is depicted for the affective condition high in appetitive motivation (bright gray circles) and the control condition (dark gray circles). Error bars depict ±1 SEM.

Effects of arousing, pleasant affect on VWM for emotional faces were tested using a repeated measures ANOVA for sensitivity *d*′ (see **Figure 6**) with emotional expression (angry, fearful, happy, neutral) and mood condition (neutral, positive arousing) as within-subject variables. Results indicated a significant interaction between the two within-subjects variables emotional expression and mood condition, *F*(3,87) = 12.689, *p* < 0.001, η^2^ = 0.304. Neither emotional expression, *F*(3,87) = 2.253, *p* = 0.088, nor mood condition, *F*(1,29) = 2.700, *p* = 0.111, showed significant main effects. To further clarify how VWM for emotional faces changed within each mood condition, we applied repeated-measures ANOVAs with emotional expression (angry, fearful, happy, neutral) as within-subject variable separately for the neutral and negative arousing condition. Considering solely the neutral condition, there was a significant effect of emotional expression, *F*(3,87) = 13.666, *p* < 0.001, η^2^ = 0.320. Here, angry looking faces showed significantly higher sensitivity than anxious (*p* < 0.001), happy (*p* < 0.001), or neutral faces (*p* = 0.003). In contrast to the neutral condition, there were no significant results in the positive arousal condition, *F*(3,87) = 1.386, *p* = 0.252. Comparing the two mood conditions for each emotion separately, planned paired-samples *t*-tests showed a significant decrease of sensitivity for angry looking faces *t*(29) = 3.596, *p* = 0.001, as well as higher *d*′ for anxious, *t*(29) = –4.149, *p* < 0.001, and happy faces, *t*(29) = –2.512, *p* = 0.018, in the positive arousal condition in contrast to the control condition. No difference was observed for neutral faces, *t*(29) = –1.476, *p* = 0.151. **Table 2** describes group means for sensitivity, illustrated in **Figure 6**.

**Table 2:** Mean *d*′ scores for the mood conditions of Experiment 2.

	Neutral			Positive arousal		
Expression	Hit rates	False alarms	Sensitivity *d*′	Hit rates	False alarms	Sensitivity *d*′
Angry	0.67 (0.03)	0.18 (0.03)	1.59 (0.12)	0.64 (0.03)	0.25 (0.04)	1.20 (0.10)
Fearful	0.63 (0.03)	0.28 (0.03)	1.02 (0.09)	0.66 (0.02)	0.19 (0.03)	1.42 (0.09)
Happy	0.64 (0.04)	0.27 (0.02)	1.10 (0.12)	0.66 (0.03)	0.22 (0.03)	1.35 (0.10)
Neutral	0.63 (0.03)	0.25 (0.02)	1.13 (0.10)	0.62 (0.03)	0.19 (0.02)	1.32 (0.11)

*SE in parentheses.*

Considering alterations of hit rates or false alarms, respectively, we applied distinct repeated-measures ANOVAs with emotional expression (angry, fearful, happy, neutral) and mood condition (neutral, positive arousing) as within-subject variables for both measures. Results for hit rates indicated no interaction effect between the within-subject variables, *F*(3,87) = 1.807, *p* = 0.152, and no main effects of the displayed emotion, *F*(3,87) = 1.058, *p* = 0.371, or the mood conditions, *F*(1,29) = 0.010, *p* = 0.921. Separate ANOVAs for each mood condition revealed no significant differences across emotional expressions, neither in the neutral condition, *F*(3,87) = 1.366, *p* = 0.259, nor in the positive arousal condition, *F*(3,87) = 1.332, *p* = 0.269. Planned *t*-tests for testing differences between the mood conditions did not show significant effects either.

Compared with this, analysis of the false alarm rates showed a significant interaction between both within-subject variables, *F*(3,87) = 8.893, *p* < 0.001, η^2^ = 0.235, and a main effect of mood condition, *F*(1,29) = 4.265, *p* = 0.048, η^2^ = 0.128, whereas the difference between the emotional expressions did not reach significant levels, *F*(3,87) = 1.230, *p* = 0.304. Comparing both the positive arousal and control condition directly for each separate emotional expression, paired-samples *t*-tests indicated a significant increase of false alarms for angry looking faces in the positive arousal condition, *t*(29) = –2.579, *p* = 0.015, whereas both anxious, *t*(29) = 4.075, *p* < 0.001, and happy faces, *t*(29) = 2.383, *p* = 0.024, showed a significant decrease of false alarm rates. There was also a slightly significant decrease of false alarms for neutral looking faces, *t*(29) = 1.966, *p* = 0.059. We observed a significant main effect of emotional expressions in the neutral condition, *F*(3,87) = 7.848, *p* < 0.001, η^2^ = 0.213. Both anxious (*p* = 0.003) and happy faces (*p* = 0.001) showed a significantly higher rate of false alarms. Compared to angry expressions, the increase of false alarms rates for neutral expressions was barely significant (*p* = 0.075). In contrast, there was only a slightly significant main effect in the positive arousal condition, *F*(3,87) = 2.279, *p* = 0.085, η^2^ = 0.073, with no significant differences between the emotional faces revealed by *post hoc* comparisons. All other multiple comparisons not mentioned here were non-significant (at least *p* > 0.200).

### Discussion

Experiment 2 demonstrates comparable effects of affective states on VWM for emotionally expressive faces as shown in Experiment 1. Low arousing affective states lead to an angry-face specificity in VWM (Jackson et al., 2009), whereas highly appetitive states result in an equal representation of all faces in VWM. Here again, the effect was mainly driven by an increase of false alarms for angry looking faces and a reduction thereof for anxious or happy expressions. As in Experiment 1, this led to an overall alignment of *d*′ for emotionally expressive faces in the positive arousal condition. Since both affective states high in appetitive motivation (e.g., desire) as well as affective states high in defensive motivation (e.g., fear) induce the same modulation of VWM, we suggest that instead of valence, motivational intensity seems to be the crucial dimension causing a switch from specificity to sensitivity in VWM processing.

## General Discussion

Consistent with our predictions, both experiments indicate a shift from specifically enhanced processing of social cues associated with threat in VWM toward an unspecific sensitivity to the presented stimuli by affective states of high motivational intensity. Experiment 1 showed specifically enhanced memory performance for angry looking faces in VWM in the neutral condition. Inducing a highly motivational affect by exposure to negatively arousing scenes led to an equal representation of all encoded stimuli in VWM without any specific prioritization for threat-related faces. Experiment 2 showed that even under the exposure to highly appetitive scenes, there is a flexible change in resource allocation for faces in VWM. As expected, the anger superiority effect disappeared in favor of an equally sensitive representation of the stimuli. In both experiments stimulus-driven anger-specificity is traceable to elevated affective salience of anger expressions, which signal interpersonal aggression. An arousing affective state, and thus mood induction, acts as perceiver-based context for face memory, which shifts resource allocation in VWM from stimulus-driven specificity to equalized sensitivity. Thus, affective states high in motivational intensity override stimulus-driven effects on VWM. The present study is the first that proves evidence for a systematic change in resource allocation in VWM during affective states by means of effective mood induction. Our results demonstrate that cognitive processing of emotional cues is subject to a flexible prioritization depending on context and state, according to the immediate goals of an organism (Cunningham et al., 2008; Cunningham and Brosch, 2012). Two pivotal findings of this study require further explanation: firstly, the specifically improved memory performance for angry looking faces compared to other emotionally expressive faces in a neutral affective state; secondly, the contextual change of memory performance for emotional faces in an affective state high in motivational intensity.

Attention and working memory interact closely and share an overlapping neural basis (Awh and Jonides, 2001; Chun, 2011; Chun et al., 2011). Specifically enhanced memory performance for angry looking faces may be a result of a threat or relevance detection mechanism, which is also responsible for the efficient detection of emotionally arousing faces in visual search (Lundqvist et al., 2014). Even though only few (three or four) chunks can be represented in VWM (Cowan, 2001), it remains unclear whether this limit is fixed or variable along with stimulus properties. Studies using the delayed-estimation protocol confirm the view of this storage as a limited resource (Ma et al., 2014) and demonstrate that precision in VWM is continuous and variable across items and trials (van den Berg et al., 2014). Equal to the allocation of attentional resources, storage capacity of VWM can be distributed in a flexible way according to visual salience or top–down goals such as cueing of stimuli qualities (Gorgoraptis et al., 2011; Melcher and Piazza, 2011; Pedale and Santangelo, 2015). Both experiments of this study prove that in a neutral affective state emotional salience, like perceptual salience (Fine and Minnery, 2009) and novelty (Mayer et al., 2011), play a key role determining resource allocation for visual cues in working memory. Emotionally salient cues such as angry looking faces mobilize additional resources in VWM and thus are represented more precisely and recognized more accurately. Enhanced representation of angry expressions does neither occur due to an increased selective attentional allocation to threatening faces, nor at the expense of other items (Thomas et al., 2014), but rather results from a specific reinforcement of one or more represented emotionally salient items (Vuilleumier, 2005; Pessoa, 2008; Pessoa and Adolphs, 2010; Todd et al., 2012b). Furthermore, early bottom-up processing in visual areas is influenced by an organism’s inner states (Rossi and Pourtois, 2012). Hence, visual processing of environmental cues is not a fixed and unalterable process, but sensitive to stimulus-driven (Vuilleumier, 2005) and state-dependent influences (Mather and Sutherland, 2011). We argue that in this way, arousing affective states act as perceiver-based context and modulate the stimulus-driven effects of angry expressions on representations in VWM in both experiments of this study. The assumption that all stimuli occurring in an affective state are rated as emotionally salient could account for the different results between mood conditions. Thus, stimuli encoded during an affective state high in motivational intensity should be represented as accurately as emotionally salient angry face displays. However, in both experiments, memory performance in the affective condition was not enhanced compared to the neutral control condition. Hence, items memorized in the affective conditions are not remembered with the same precision as threatening faces in the neutral condition.

Altogether, affective states do not lead to a general increase in accuracy, but more likely to a change in processing the items. A possible cause could be the special configural or holistic processing of upright faces. In VWM, too, this integration or chunking of a facial feature to a holistic percept (Richler and Gauthier, 2014) leads to enhanced memory performance of upright faces in comparison to inverted faces (Curby and Gauthier, 2007). Consistent with current approaches describing VWM as a flexible resource, this effect is not attributable to a higher capacity, but rather to higher resolution of upright faces (Scolari et al., 2008; Lorenc et al., 2014). Not only stable characteristics of a face, but also dynamic emotional expressions are at least in part being processed holistically (Gupta and Srinivasan, 2009; Tanaka et al., 2012; Bombari et al., 2013). Therefore, an anger-specificity in VWM could arise through a tuned integration of an angry face feature, resulting in a more precise holistic representation (Jackson et al., 2009). However, processing of faces is not necessarily holistic. Inducing a negative affective state result in a decrease of holistic face processing (Curby et al., 2012). This finding complies with the assumption that high-motivation affective states (e.g., fear, desire) narrow cognitive scope (Gable and Harmon-Jones, 2010a) and therefore result in a more analytic, feature-by-feature based processing of faces. Focusing attention to stimulus properties when viewing whole emotional images leads to affective filtering (Todd et al., 2012a) and overrules affective responses to emotional salient stimuli (Everaert et al., 2014). In the present study, affect-induced narrowing of the cognitive scope may lead to feature-based face processing and disrupt holistic processing of emotional expressions. Such an interruption may direct focus on specific facial features instead of a holistic overall appearance and thus inhibit the indirect influence of affective information originating from an emotionally expressive face. Since the anger-specificity in VWM may be driven by affective salience, the consequence of a filtering of the emotional content by affective states high in motivational intensity would be aligned processing of all emotionally expressive faces. This would correspond to the found changes between mood conditions in both experiments. An enhanced representation of angry expressions could be prevented by affective filtering due to narrowed cognitive scope and may lead to an equally sensitive representation of items in VWM, regardless of the emotional content.

Alternatively, instead of a shift in cognitive scope by high motivational affects, the resource limiting effect of arousing affective states could underlie the disappearance of the anger superiority effect in VWM. In this experimental design, emotional information derived from the faces to be remembered has an implicit influence on VWM performance since it is not the emotional expression that is being remembered explicitly, but the face identity itself. Recent evidence could show that an increase of working memory load eliminates specific processing of threatening faces (Van Dillen and Koole, 2009; Van Dillen and Derks, 2012). Therefore, enhanced storage of angry looking faces might as well depend on the availability of cognitive resources, conversely a reduction of resources in VWM by arousing affective states might eliminate threat-related memory bias. Exposure to affect-inducing situations results in altered performance in VWM (Schoofs et al., 2008; Qin et al., 2009). If affective states high in motivational intensity lead to a restriction of available resources in VWM, sensitivity as a measure of memory performance should decrease in the experimental conditions of Experiments 1 and 2 compared to the control condition. However, results show no significant decrease in sensitivity between the mood conditions. More recent findings estimate the capacity of VWM for faces slightly higher than the four items to be encoded in this study (Lorenc et al., 2014). Thus, additional limitation of resources by a distracting mood induction may inhibit this indirect effect of angry expressions not relevant to the task without impairing memory performance for the four faces. Limited resources due to arousing affective states may disrupt the extraction of emotional content of faces and lead to aligned sensitivity for all emotionally expressive faces in the affective condition. Overall, our findings prove strong evidence for a state-dependent flexibility of resource allocation in VWM for affective salient stimuli.

On a neurophysiological level, the modulation of VWM for emotionally expressive faces by arousing affective states might be driven by an arousal-induced modulation of catecholamine systems (Chamberlain et al., 2006; Berridge, 2008; Sara, 2009; Hermans et al., 2011, 2014; Sara and Bouret, 2012; Avery et al., 2013). Distinct norepinephrine and dopamine activity states coordinate sensory and mnemonic processing (Goto et al., 2007; Arnsten, 2011; Cools and D’Esposito, 2011). Furthermore, phasic norepinephrine release leads to an enhanced representation of emotional cues in the visual cortex through an interaction with affective salience networks (Waterhouse et al., 1998; Todd et al., 2012b; Markovic et al., 2014). Such a prioritization of visual affective cues could underlie the enhanced VWM for angry looking faces. During highly arousing affective states, tonic release of both catecholamines is increased (Rajkowski et al., 1994; Berridge, 2008; Arnsten, 2011) and thereby disrupts phasic signaling (Valentino and Wehby, 1988; Berridge and Waterhouse, 2003; Arnsten, 2011), resulting in a state of indiscriminate cognitive processing. Hence, inducing affective states high in motivational intensity, as applied in both of our Experiments, should result in high tonic release. This disrupts a short-duration burst of catecholamine activity and may result in an equally sensitive representation of faces in VWM instead of enhanced processing of angry faces. Indeed, affective arousal leads to a rapid extraction of the gist (Qin et al., 2012) and to faster responses at the cost of an increasing rate of false alarms (Duncko et al., 2009). In line with these assumptions, the results of both experiments indicate no generally enhanced storage of all stimuli under highly arousing affective states, but rather suggest an altered mode of processing which disrupts the selectively enhancing influence of threat-related cues. In fact, recent neuropharmacological evidence supports the concept that there is a strong relationship between classification of angry faces (Lawrence et al., 2007) as well as enhanced VWM for angry looking faces (Subramanian et al., 2010) and dopaminergic function.

Contrary to our results, Jackson et al. (2009) excluded arousal as an influencing factor of the anger superiority effect in VWM, because they found no effect of quiet or loud sounds presented parallel to emotionally expressive faces. Unlike angry looking faces or emotional scenes (e.g., mutilation, weapons, erotica), loud sounds *per se* are neither affectively salient nor resulting in a state of high motivational intensity, therefore effects remain absent. Schaller et al. (2003) were able to prove that in the absence of ambient light, negative threat-associated attributions toward out-group members are being stimulated. In different designs (watching a film clip depicting a female being stalked by a serial killer, visualizing being a soldier under attack by hostile forces), Becker et al. (2010) specifically activated self-protection goals, which led to enhanced coding efficiency for outgroup male faces groups, but not for female or same-race male faces. Also, Becker et al. (2014) demonstrated an enhanced benefit for angry looking faces after participants were primed for self-protective goals (visualizing being alone in a house with an intruder). All mentioned results show that a sense of self-protection causes, or at least reinforces, enhanced processing of threatening faces. In contrast, the results at hand show that exposure to highly arousing images leads to unspecific sensitivity in VWM and eliminates the benefit for angry expressions. These differences might arise from the methods and experimental protocols used. In contrast to contradicting findings, we used highly arousing IAPS images in the present study. Similar to highly arousing scenes involving violence (e.g., van Marle et al., 2009), photographs of mutilations or explicitly erotic scenes could trigger more intense affective arousal responses than the mentioned experimental protocols. Furthermore, presenting highly arousing IAPS scenes throughout the entire task processing might lead to a longer-lasting and more persistent manipulation of affective states than a singular mood induction before task processing. Indeed, there is evidence that the physiological and subjective responses differ during and after stress induction (Hellhammer and Schubert, 2012).

Previous studies showed an influence of sex and ethnicity on face perception and memory (Becker et al., 2010, 2014). Future research may address the influence of affective states as perceiver-based context for face processing regarding those characteristics. In contrast to more recent investigations, our study explores resource allocation using signal detection measures. Recently published studies employ the delayed estimation protocol in order to examine resource allocation in VWM (Wilken and Ma, 2004; Luck and Vogel, 2013). This paradigm allows to measure the precision of representations in VWM. Future studies could use the delayed estimation protocol to examine resource allocation in VWM for emotionally expressive faces both under neutral conditions as well as during affective states of varying intensity. While our study focuses on VWM for emotionally expressive faces, further research should explore the generalizability of an affect-induced switch from specificity to sensitivity in working memory processing to other stimulus categories and other sensory modalities.

## Conclusion

Our study is the first one to prove evidence for a systematic shift in resource allocation in VWM by affective states of high motivational intensity. Our results present affective states as influential perceiver-based context (Barrett et al., 2011), which modulate the prioritization of representations in VWM. Angry expressions signal aggressive intentions of social encounters. In default mode, a particularly enhanced representation of an aggressive conspecific is of ecological importance in order to prevent conflicts or respond to them quickly, avoiding potential costs of an organism’s fitness (Duntley, 2005; Haselton and Nettle, 2006). Exposure to cues critical for survival, such as mutilations, erotica, or human violence, elicits affective states associated with appetitive or defensive behavioral preparedness (Löw et al., 2008; Bradley et al., 2012; Miskovic and Keil, 2014). In such a state, angry expressions lose their specific signal value. Furthermore, when expecting a biologically significant event, an organism anticipates the conspecific’s intention as being relevant for its own defensively or appetitively determined goals, regardless of the current expressive behavior. Therefore, from a functionalist perspective it is adaptive to represent all conspecifics occurring in this context rapidly, confined to the most essential attributes and with equal acuity. This supports the notion of VWM as a functionally designed cognitive system, which enables streamlined information processing along current demands of the environment (Nairne, 2010; Nairne and Pandeirada, 2010; Cosmides and Tooby, 2013). An interaction between catecholaminergic activity and affective salience networks to tune emotionally salient representations in sensory areas could account for this flexible modulation of information processing (van Marle et al., 2009; Todd et al., 2012b). Our findings mark a step forward in the understanding of the modulation of cognitive processing during affectively arousing situations, whereby those affect-sensitive cognitive mechanisms may play a crucial role in the pathogenesis of stress-related disorders.

## Conflict of Interest Statement

The authors declare that the research was conducted in the absence of any commercial or financial relationships that could be construed as a potential conflict of interest.
